# Supplementary material for: Cryo-EM structure of coronavirus-HKU1 haemagglutinin esterase reveals architectural changes arising from prolonged circulation in humans
Source: Nat Commun. 2020 Sep 16;11:4646. doi: 10.1038/s41467-020-18440-6 (PMC7495468; doi:10.1038/s41467-020-18440-6)
Supplement: Supplementary file 1 — Supplementary Information [file 41467_2020_18440_MOESM1_ESM.pdf]

Supplementary Information

Cryo-EM structure of coronavirus-HKU1 haemagglutinin esterase reveals architectural changes arising from prolonged circulation in humans

Hurdiss et al.

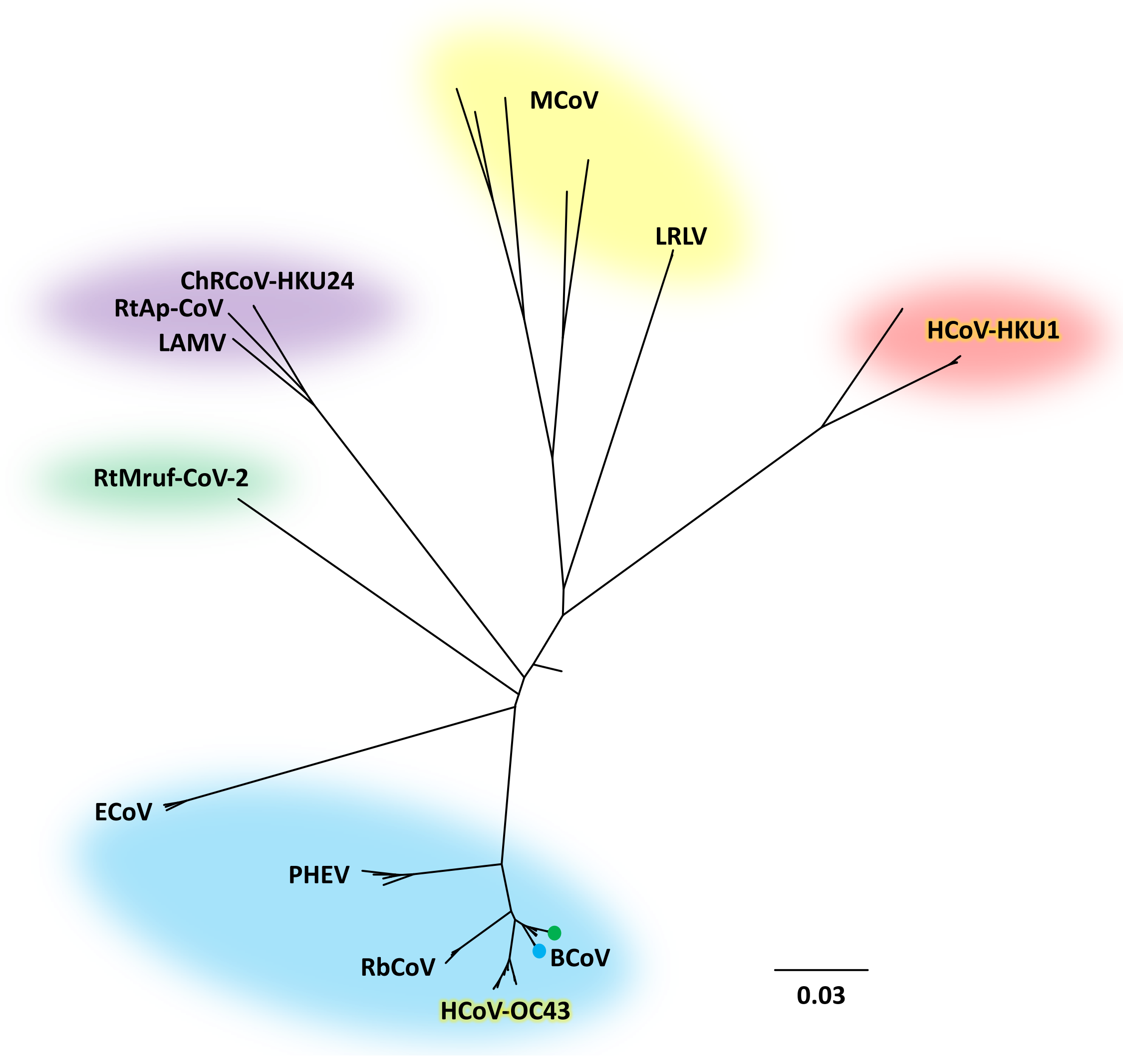

Supplementary Figure 1: Rooted Neighbour-joining tree based on embecovirus HE sequences in the NCBI database (n = 55), with 100% bootstrap support for all major branches. Evolutionary distances were computed using the Maximum Composite Likelihood method in MEGAX[1]. The positions of human coronaviruses HKU1 and OC43 are highlighted relative to those of animal embecoviruses, including members of the species *Betacoronavirus 1* and classical mouse hepatitis virus-type murine coronavirus (MCoV). ChRCoV-HKU24, Chinese rat coronavirus HKU24[2]; LAMV, Longquan Aa mouse coronavirus[3]; LRLV, Longquan Rl rat coronavirus (Wang et al., 2015); RtAp-CoV, Rodent coronavirus isolate RtAp-CoV/Tibet2014; RtMruf-CoV-2, Rodent coronavirus isolate RtMruf-CoV-2/JL2014[4]. The position of CRCoV and HKU23, recent split-offs from BCoV, are indicated by a blue and green dot, respectively

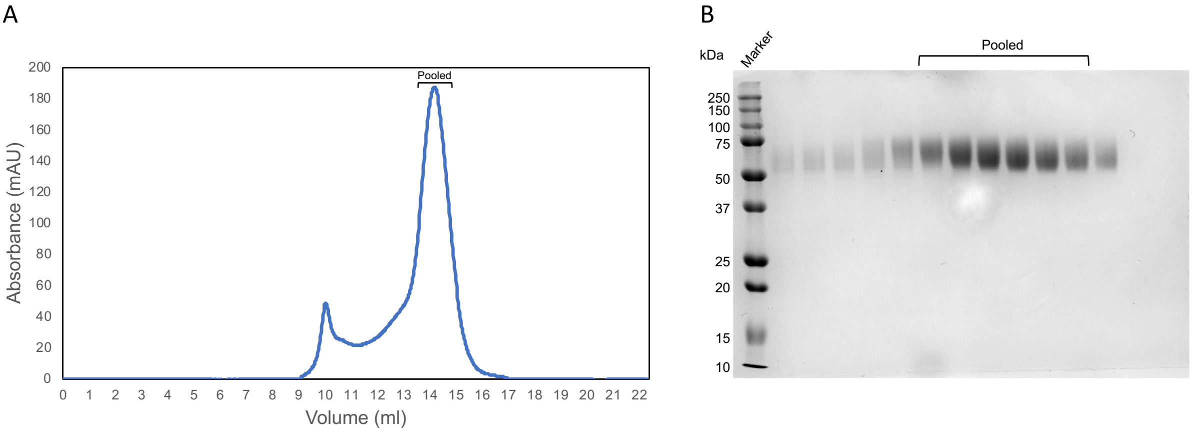

Supplementary Figure 2: Purification of HKU1 HE. A) Elution profile of HKU HE from a Superdex 200 10/300 GL gel filtration column. UV absorbance (mAU) is shown as a blue trace. Fractions pooled for subsequent cryo-EM analysis are indicated. (B) SDS-PAGE analysis of the fractions collected from the gel filtration run shown in (A). The molecular weight marker and pooled fractions are indicated.

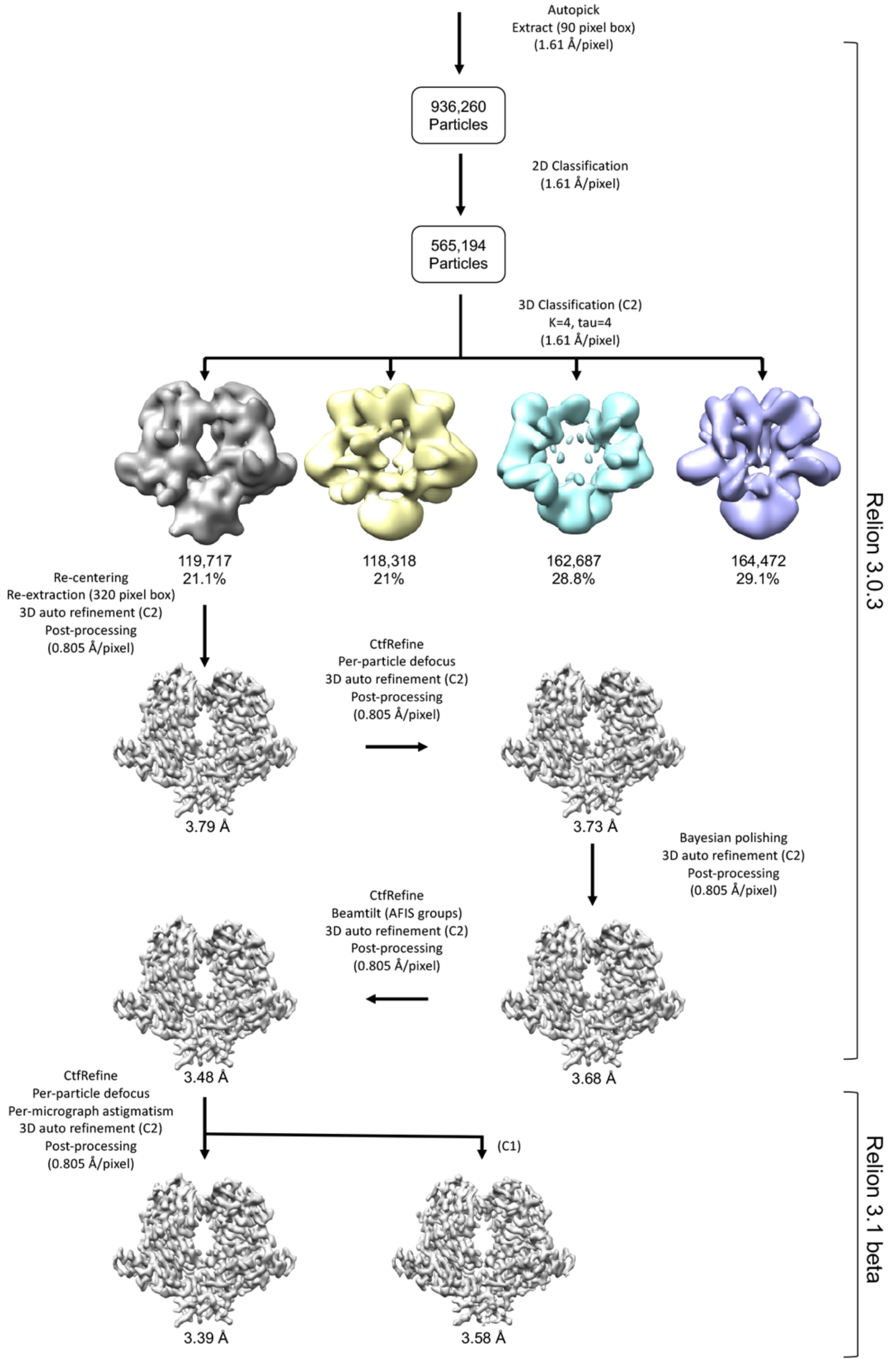

Supplementary Figure 3: Single-particle cryo-EM image processing workflow for HCoV-HKU1 HE (see methods for details).

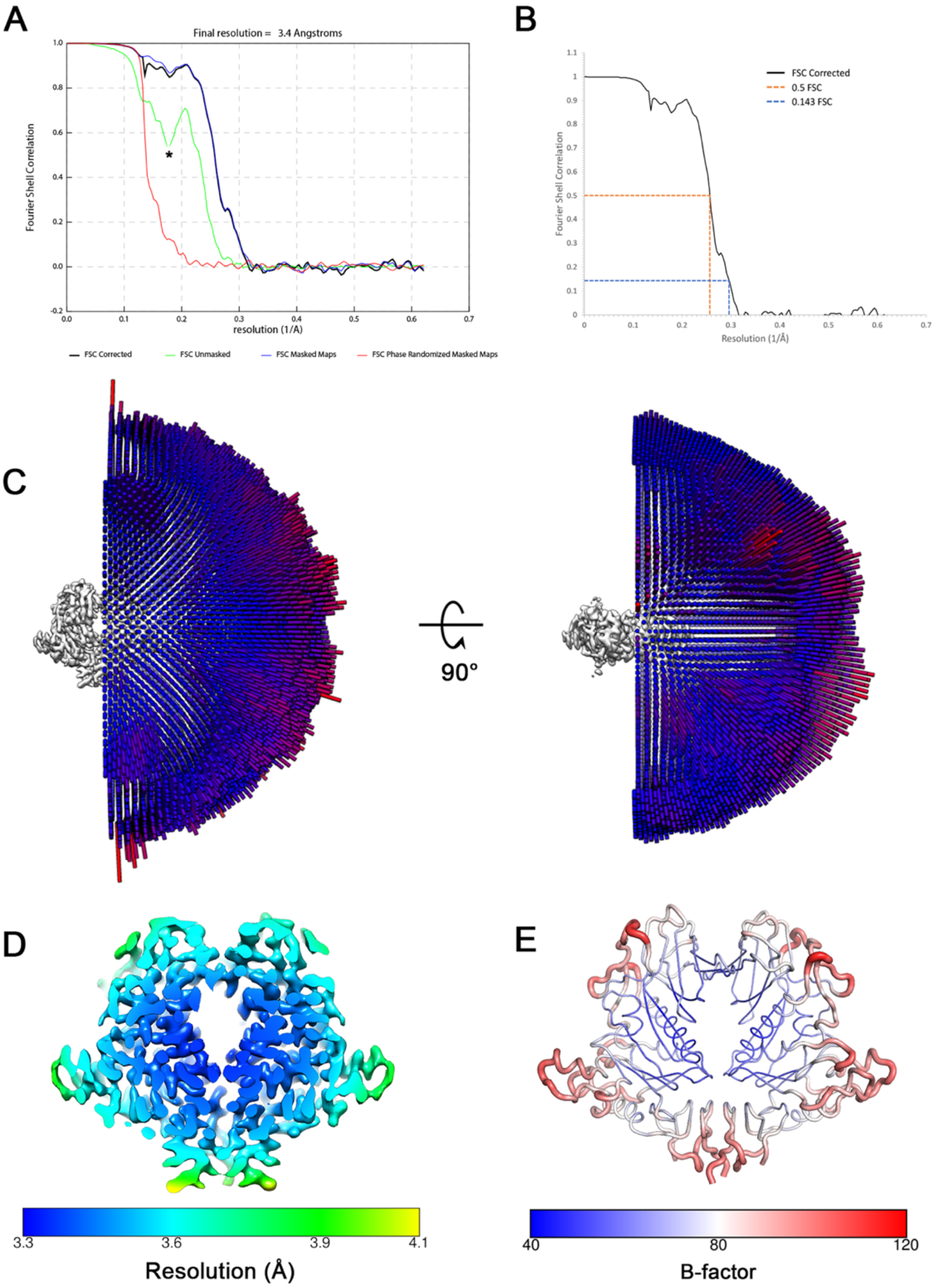

Supplementary Figure 4: A) Gold-standard Fourier shell correlation (FSC) curve generated from the independent half maps contributing to the ~3.4 Å resolution density map. A dip in the unmasked FSC at ~5.8 Å is indicated by an asterisk. This likely results from poor correlation between the disordered and heterogenous glycans at this spatial frequency. B) Corrected FSC curve with the 0.5 and 0.143 cut-off indicated by orange and blue dashed lines, respectively. C) Angular distribution plot of the final C2 refined EM density map. D) A 10 Å slice through the final C2 refined EM density map coloured according to local resolution which was calculated in Relion3.1. E) Atomic model of the dimeric complex with residues coloured according to calculated B-factor.

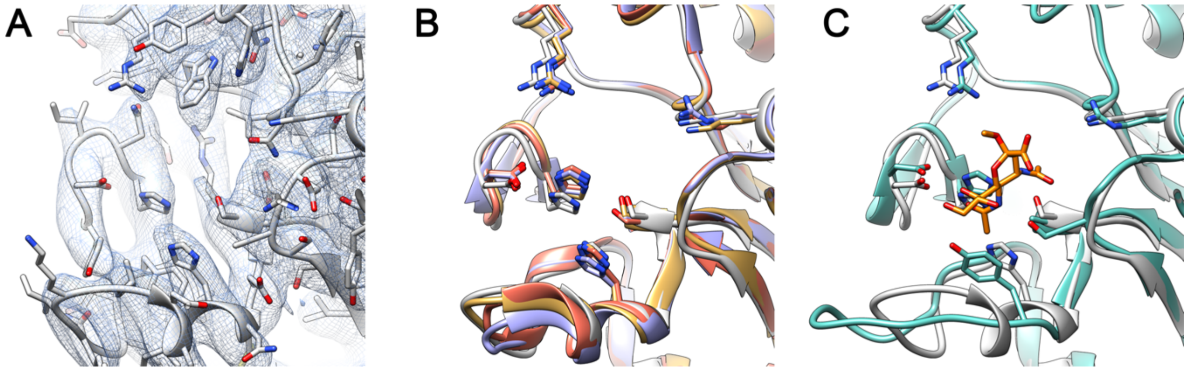

Supplementary Figure 5: A) Zoomed-in view of the HKU1 HE esterase domain active site. EM density is shown as a blue mesh and the atomic model is coloured grey. B) Overlay of the HE esterase domain active-site of HKU1 HE, BCoV (PDB ID:3CL5), MHV-DVIM (PDB ID:5JIF), and OC43 (PDB ID:5N11) coloured grey, purple, gold and red, respectively. Residues which make up the active-site are shown as sticks. C) Overlay of the HE esterase domain active-site of HKU1 HE and MHV-NJ (PDB ID:5JIL) with 4-N-Acetyl sialic acid, present in the latter structure, coloured orange.

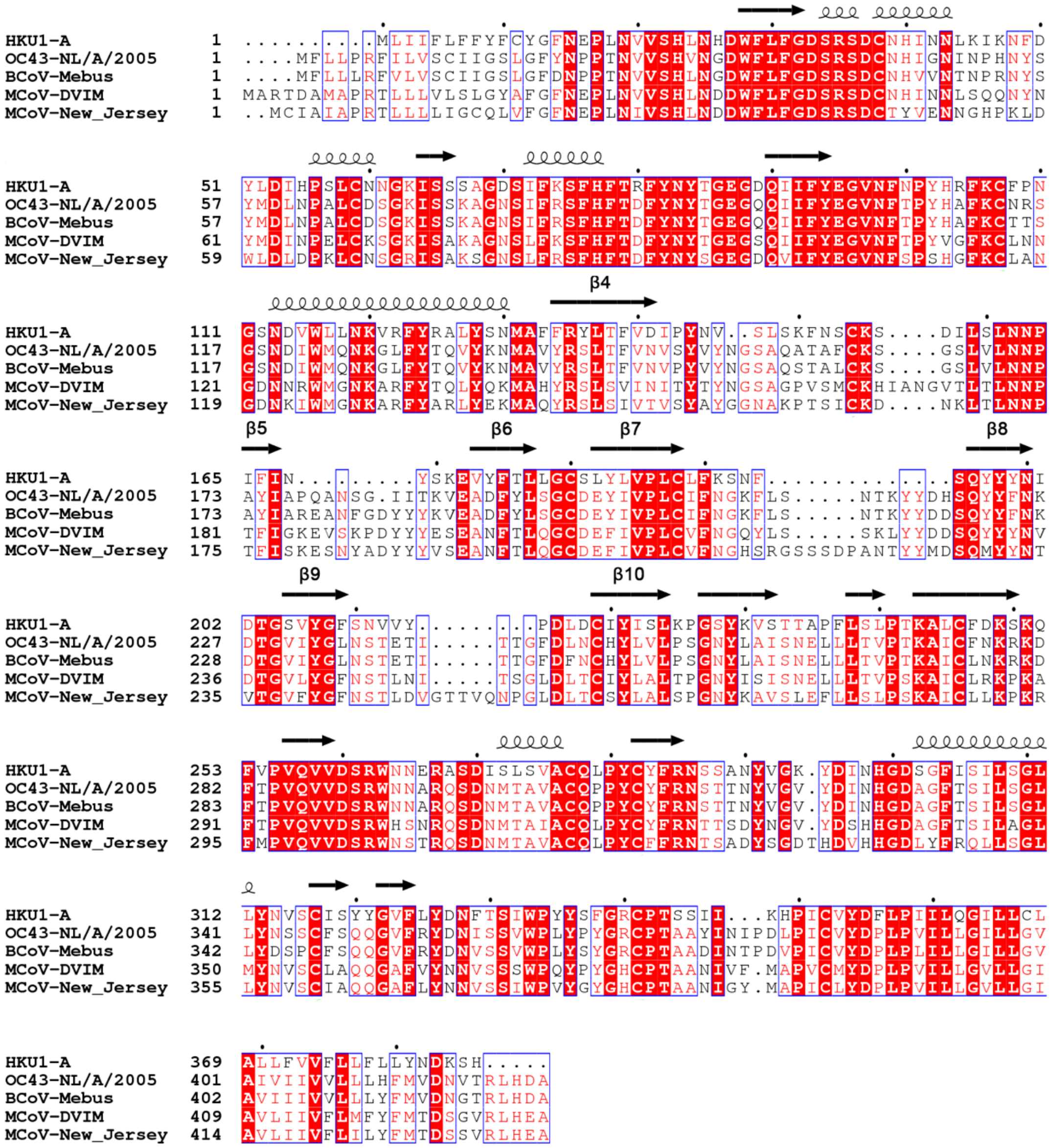

Supplementary Figure 6: Multiple sequence alignment of HKU1-A (UniProt Q5MQD1), OC43-NL/A/2005 (UniProt Q4VID6), BCoV-Mebus (UniProt P15776), MCoV-DVIM (UniProt O92367) and MCoV-NJ (UniProt Q3HS77) HE amino acid sequences. Beta strands which flank loop deletion sites are labelled. The sequence alignment was performed using Clustal Omega[5] and the image was generated by ESPript 3.01[6] . The secondary structure assignment, based on the HKU1-A cryo-EM structure, is shown.

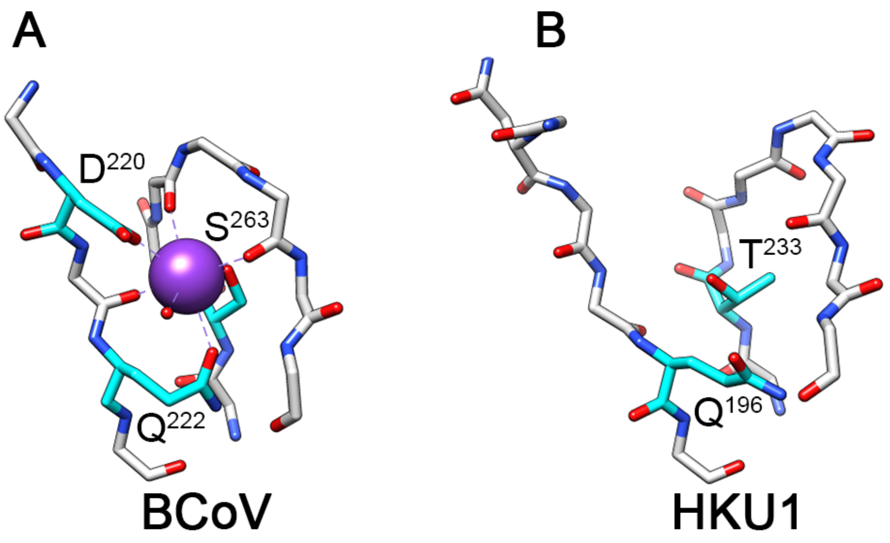

Supplementary Figure 7: A) Stick representation of the BCoV-Mebus (PDB ID:3CL5) lectin domain metal binding site, with the potassium ion shown as a purple sphere and coordinating sidechains coloured cyan. B) Equivalent view as shown in A for HKU1-A HE.

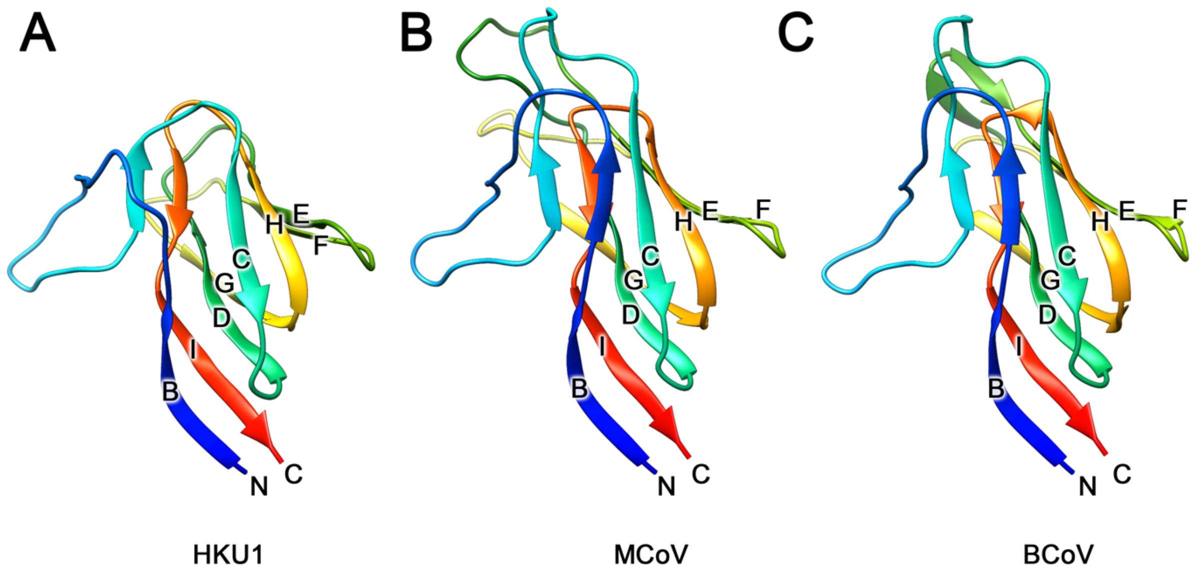

Supplementary Figure 8: Cartoon representation of the HE lectin domain from (A) HKU1-A, (B) MHV-NJ (PDB ID:5JIL) and (C) BCoV-Mebus (PDB ID:3CL5). Beta strands are shown in chainbow coloring and labeled according to the historical jelly roll nomenclature.

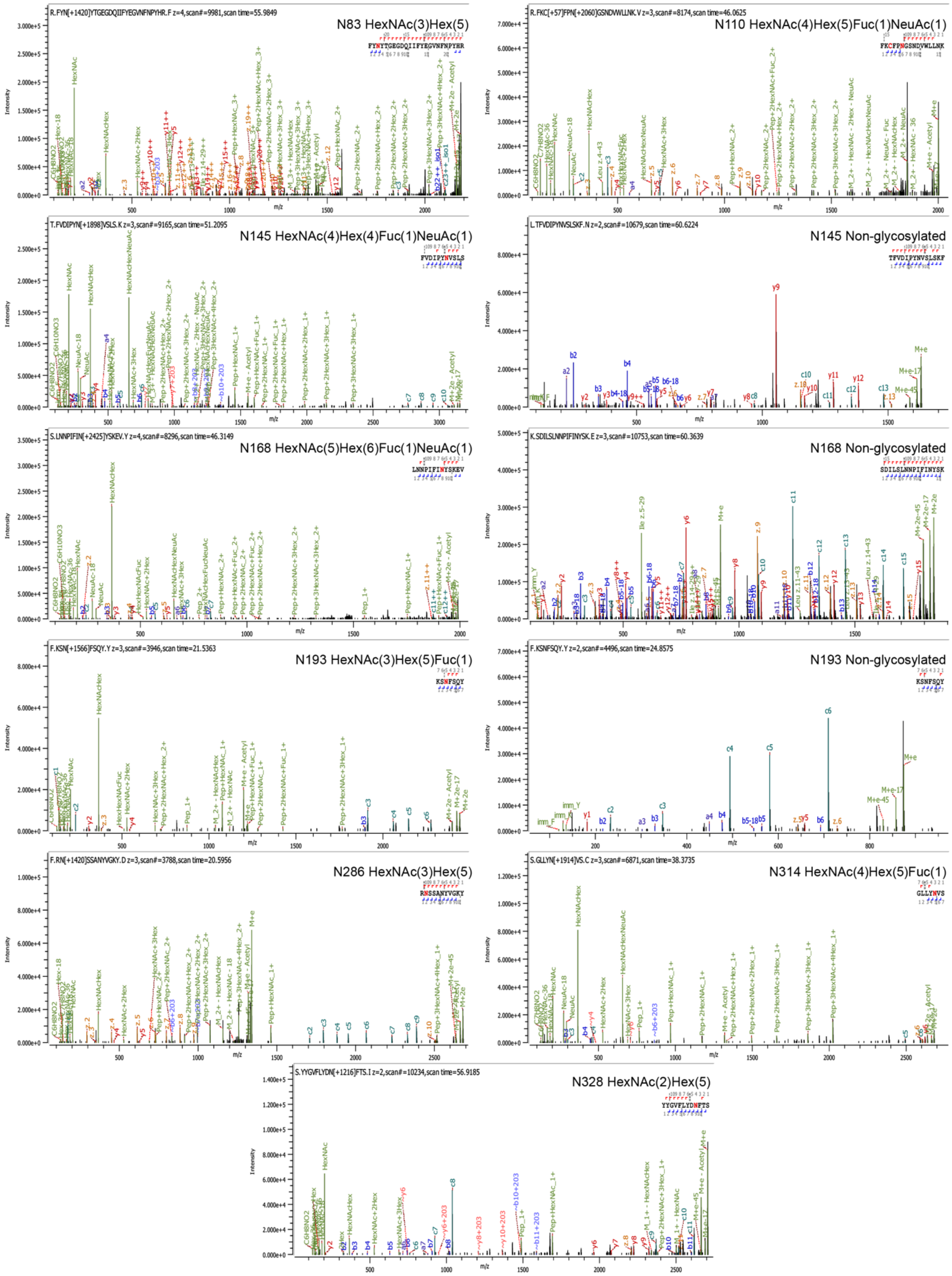

Supplementary Figure 9: Representative MS/MS spectra supporting Byonic identifications per glycosite.

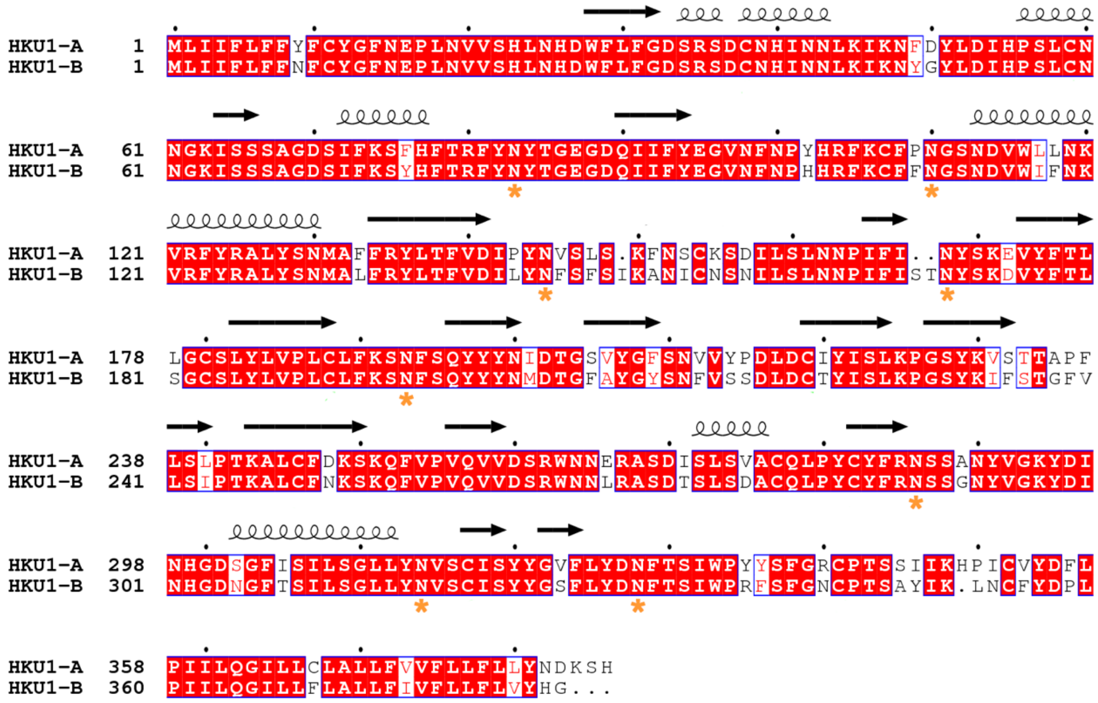

Supplementary Figure 10: Alignment of representative HKU1-A (UniProt Q5MQD1) and HKU1-B (UniProt Q14EB1) HE amino acid sequences. The eight conserved N-linked glycosylation sites are indicated with asterisks. The sequence alignment was performed using Clustal Omega[5] and the image was generated by ESPript 3.01[6]. The secondary structure assignment, based on the HKU1-A cryo-EM structure, is shown.

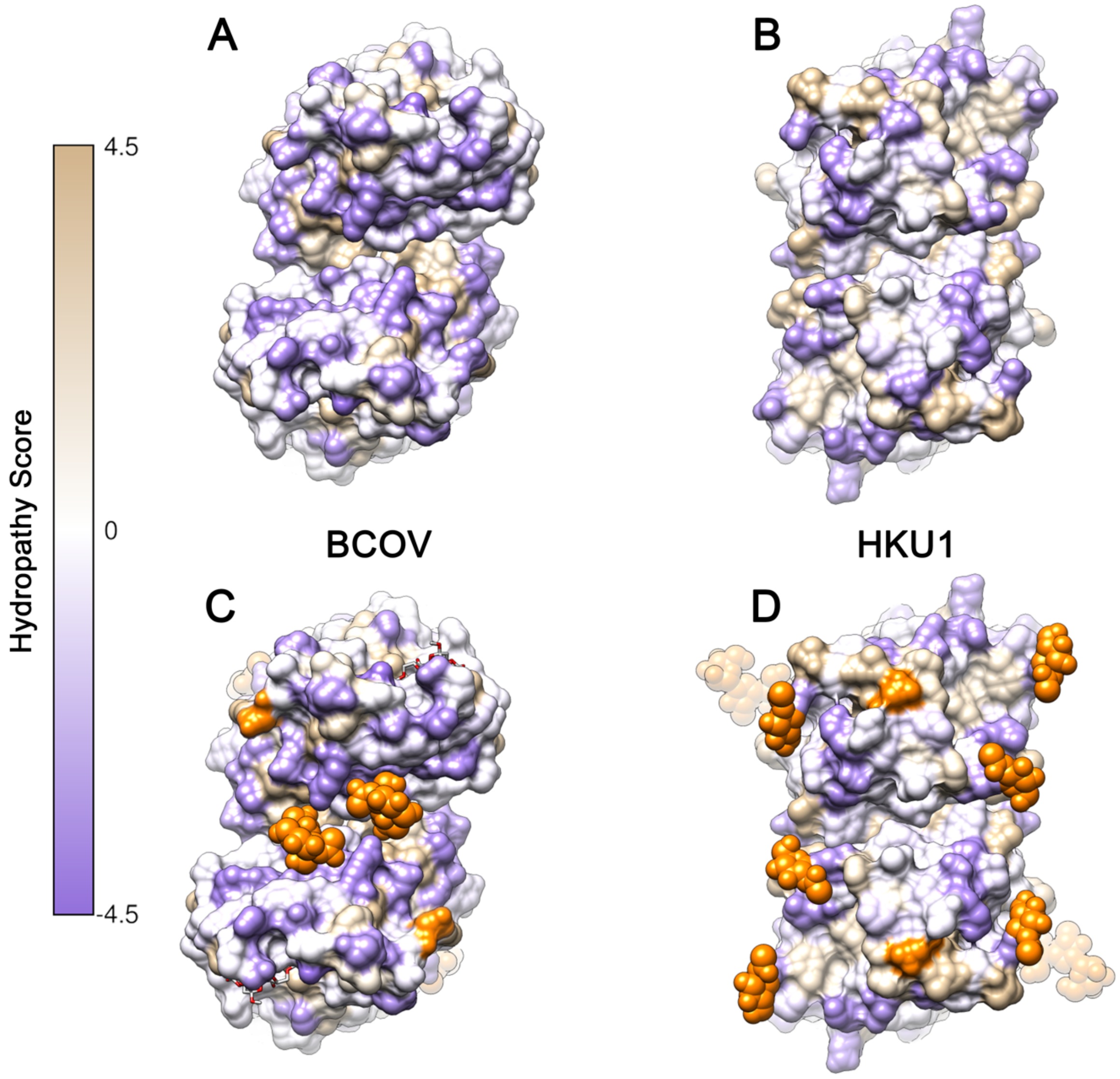

Supplementary Figure 11: A) Top down view of the dimeric BCoV-Mebus (PDB ID:3CL5) and (B) HKU1-A HE lectin domains, shown as a surface representation and coloured according to the Kyte-Doolittle scale, where the most hydrophobic residues are coloured tan and the most hydrophilic residues are coloured purple. C-D) As shown in A and B with N-glycosylation sites coloured orange and, if modelled, depicted as spheres. For BCoV, bound sialic acid is shown in stick representation and coloured white.

Supplementary Table 1: Data collection, image processing and refinement information.

**Data Collection**	
Microscope	Titan Krios G4
Voltage (keV)	300 keV
Nominal magnification	96,000x
Exposure navigation	Image shift
Movie acquisition rate	~238 per hour
Detector	Falcon 4
Calibrated pixel size (Å)	0.805
Cumulative exposure (e/Å^2)	40
Dose rate (e/pixel/sec)	5
Underfocus range (μm)	1 to 2.5 (0.25 increments)
Micrographs collected	6029
**Reconstruction**	
Final particles (no.)	119,717
Symmetry	C2
B-factor (Å^2)	-122
**Resolution (Å)**	
FSC 0.5 (masked)	3.89
FSC 0.143 (masked)	3.39
Resolution range (local)	3.3-4.8
Angular accuracy	1.58
**Refinement**	
Protein residues/atoms	664/5414
N-glycans/atoms	14/440
**Resolution (Å)**	
FSC 0.5	3.5
**Map correlation coefficient**	
Mask	0.8
Volume	0.79
Peaks	0.70
**R.M.S. deviations**	
Bond Lengths (Å)	0.007
Bond Angles (°)	0.972
**MolProbity**	
Overall score	1.79
Clashscore	4.49
Ramachandran outliers (%)	0
Ramachandran favoured (%)	89.55
Rotamer outliers (%)	0.33
C-beta outliers	0
**EMRinger Score**	4.7
**Privateer**	
Wrong anomer	0
Wrong configuration	0
Unphysical puckering amplitude	0
In higher-energy conformations	0

Supplementary Table 2: Human codon-optimised sequence for HKU1-A HE.

Gene Name	Sequence
HKU1-A HE	CTAGCCTTCAACGAGCCCCTGAATGTGGTGAGCCACCTGAACCACGACTGGTTCCTG<br>TTCGGCGATAGCCGCAGCGACTGCAACCACATCAACAACCTGAAGATCAAGAATTTC<br>GACTACCTGGACATCCACCCCAGCCTGTGCAACAACGGCAAGATCAGCTCCAGCGC<br>CGGAGACAGCATCTTCAAGAGCTTCCACTTCACTCGCTTCTACAACTACACCGGCGA<br>GGGCGACCAGATCATCTTCTACGAGGGCGTGAACTTCAATCCCTACCACCGCTTCAA<br>GTGCTTTCCCAACGGCAGCAACGACGTCTGGCTGCTGAACAAGGTCCGCTTCTACCG<br>GGCCCTGTACAGCAACATGGCCTTCTTCCGCTACCTGACGTTCGTGGACATTCCCTAC<br>AACGTGAGCCTGTCCAAGTTCAACAGCTGTAAGTCCGACATCCTGTCCCTGAACAAC<br>CCCATCTTCATCAATTACAGCAAGGAGGTGTACTTCACCCTGCTCGGCTGCAGCCTG<br>TACCTGGTCCCTCTGTGCCTGTTCAAGAGCAACTTCAGCCAGTACTATTACAACATCG<br>ACACCGGCAGCGTGTACGGGTTCAGCAACGTGGTGTATCCCGACCTGGACTGCATC<br>TACATCAGCCTGAAGCCCGGCTCCTACAAGGTGTCCACCACTGCGCCTTTCCTGTCCC<br>TGCCCACGAAGGCCCTCTGCTTCGATAAGAGCAAACAGTTCGTGCCCGTACAGGTC<br>GTGGACAGCCGCTGGAACAATGAGCGGGCCAGCGACATCTCCCTGTCCGTGGCCTG<br>CCAGCTGCCCTACTGCTATTTCCGCAACAGCTCTGCCAACTACGTGGGCAAGTACGA<br>CATCAACCACGGCGATAGCGGCTTCATCAGCATCCTGAGCGGCCTGCTGTACAACGT<br>GAGCTGCATCAGCTACTATGGCGTCTTCCTGTACGACAACTTCACCTCCATCTGGCCC<br>TACTACAGCTTCGGCAGGTGTCCCACCAGCTCTATTATCAAGCACCCCATCTGCGTGT<br>ACGACTCG

Supplementary Table 3: Overview of common N-glycoforms shared at the majority of sites with predicted structures.

Present at No. of glycosites	Predicted components	Predicted structure
6 out 8	HexNac4Hex5Fuc1	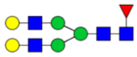
6 out 8	HexNac4Hex5Fuc1NeuAc1	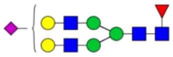
5 out 8	HexNac3Hex5Fuc1	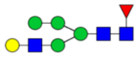
5 out 8	HexNac3Hex6	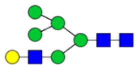
5 out 8	HexNac3Hex6Fuc1	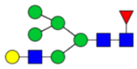
5 out 8	HexNac4Hex4Fuc1	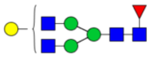
5 out 8	HexNac5Hex4Fuc1	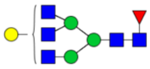
5 out 8	HexNac5Hex6Fuc1NeuAc1	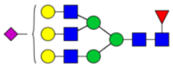
4 out 8	HexNac2Hex5	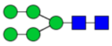
4 out 8	HexNac3Hex5	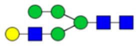
4 out 8	HexNac4Hex5	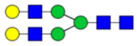
4 out 8	HexNac4Hex3Fuc1	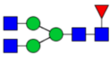
4 out 8	HexNac4Hex4Fuc1NeuAc1	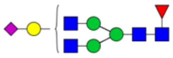
4 out 8	HexNac5Hex3Fuc1	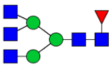
4 out 8	HexNac5Hex5Fuc1	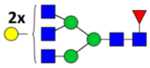

Supplementary References

1. Kumar, S., Stecher, G., Li, M., Knyaz, C. & Tamura, K. MEGA X: Molecular Evolutionary Genetics Analysis across Computing Platforms. *Mol. Biol. Evol.* **35,** 1547–1549 (2018).
2. Lau, S. K. P. *et al.* Discovery of a novel coronavirus, China Rattus coronavirus HKU24, from Norway rats supports the murine origin of Betacoronavirus 1 and has implications for the ancestor of Betacoronavirus lineage A. *Journal of Virology* **89,** 3076–3092 (2015).
3. Wang, W. *et al.* Discovery, diversity and evolution of novel coronaviruses sampled from rodents in China. *Virology* **474,** 19–27 (2015).
4. Wu, Z. *et al.* Comparative analysis of rodent and small mammal viromes to better understand the wildlife origin of emerging infectious diseases. *Microbiome* **6,** 178–14 (2018).
5. Madeira, F. *et al.* The EMBL-EBI search and sequence analysis tools APIs in 2019. *Nucleic Acids Res.* **47,** W636–W641 (2019).
6. Robert, X. & Gouet, P. Deciphering key features in protein structures with the new ENDscript server. *Nucleic Acids Res.* **42,** W320–4 (2014).
