# Supplementary material for: Cryo-EM structure of coronavirus-HKU1 haemagglutinin esterase reveals architectural changes arising from prolonged circulation in humans
Source: Nat Commun. 2020 Sep 16;11:4646. doi: 10.1038/s41467-020-18440-6 (PMC7495468; doi:10.1038/s41467-020-18440-6)
Supplement: Supplementary file 3 — Description of Additional Supplementary Files [file 41467_2020_18440_MOESM3_ESM.pdf]

Description of Additional Supplementary Files

File Name: Supplementary Data 1
Description: Overview of HKU1-HE glycoproteomics data. Full lists of identified site-specific glycoforms and their semiquantitative analysis are provided per protease dataset.

File Name: Supplementary Data 2
Description: HKU1 and BCoV HE sequences used for sequence conservation analysis.
